# Psychometric Properties of the Greek Version of the ASKAT: Assessing Teachers’ Knowledge and Attitudes Toward ADHD

**DOI:** 10.12688/f1000research.176376.1

**Published:** 2026-04-13

**Authors:** Paraskevi Boukouvala, Spyridon-Georgios Soulis, Konstantinos Kotsis, Vassiliki Siafaka

**Affiliations:** 1 Department of Speech and Language Therapy, University of Ioannina, Ioannina, Epirus, 45110, Greece; 2 Department of Primary Education, University of Ioannina, Ioannina, Epirus, 45110, Greece; 3 Faculty of Medicine, University of Ioannina, Ioannina, Epirus, 45110, Greece

**Keywords:** ADHD; Greek version of ASKAT; teachers; specific knowledge; attitudes; psychometric validation; inclusive education.

## Abstract

**Background:**

Attention-Deficit/Hyperactivity Disorder (ADHD) is a prevalent neurodevelopmental condition affecting a significant percentage of school-aged children worldwide. Teachers play a crucial role in identifying ADHD-related behaviors and fostering inclusive classroom environments. This study aimed to validate the Greek version of the ADHD-Specific Knowledge and Attitudes of Teachers (ASKAT) questionnaire and assess the knowledge and attitudes of Greek preschool and primary school teachers regarding ADHD.

**Methods:**

A national sample of 1,032 teachers from both public and private schools across Greece completed the ASKAT online. The analysis focused on the ADHD-specific knowledge and attitudes components of the tool.

**Results:**

Confirmatory Factor Analysis (CFA) supported the five-factor structure of the Attitudes Scale (SASA) (CFI = 0.961; TLI = 0.953), confirming the internal consistency of the attitude-related factors. Internal consistency was found to be acceptable for most SASA subscales (α ≥ 0.61), while the subscale “Positive Consequences of ADHD-type Behaviors” demonstrated slightly lower reliability (α = 0.57). The Knowledge Scale (SASK) also showed acceptable reliability (α = 0.656). Overall, teachers displayed adequate knowledge and generally favorable attitudes toward ADHD, with significant variations observed based on gender, education level, ADHD-related training, and teaching experience. These findings emphasize the need for continued refinement and validation of measurement tools, ensuring their relevance and reliability for use in educational settings.

**Conclusions:**

The Greek version of the ASKAT serves as a valuable instrument for assessing teachers’ ADHD-related knowledge and perceptions, laying the groundwork for focused professional development initiatives aimed at promoting inclusive practices in schools.

## Introduction

Attention-Deficit/Hyperactivity Disorder (ADHD) is among the most common neurodevelopmental disorders of childhood and is characterized by persistent inattention, impulsivity, and hyperactivity that can interfere with functioning across settings ( American Psychiatric Association, 2013; Barkley, 2015). In school contexts, ADHD-related difficulties are often reflected in reduced learning engagement, challenges in behavioral self-regulation, and peer relationship problems, with important implications for inclusive classroom practices and educational adjustment ( Barkley, 2015; Wolraich et al., 2020). Although typically identified during childhood, ADHD frequently persists into adolescence and adulthood and commonly co-occurs with learning, language, and emotional–behavioral difficulties, contributing to broader functional impact ( Rajaprakash & Leppert, 2022; Salari et al., 2023).

Epidemiological estimates suggest that ADHD affects approximately 5–8% of school-aged children worldwide ( Ayano et al., 2023). At the same time, recognition and support practices vary across educational systems, reflecting differences in awareness, referral pathways, and access to assessment and intervention services ( Kooij et al., 2019). In Greece, a national study reported a prevalence of approximately 6% among primary school children, with notable gender differences ( Giannopoulou et al., 2017). This variability highlights the importance of strengthening school-based capacity to recognize ADHD-related needs and respond with appropriate educational supports ( Wolraich et al., 2020).

Teachers are often among the first adults to observe persistent ADHD-related behaviors in everyday classroom routines and may raise initial concerns before parents or healthcare professionals ( Cueli et al., 2023). However, teachers’ responses to ADHD-related behaviors are shaped not only by knowledge of symptoms, etiology, and evidence-based interventions, but also by attitudes and beliefs that influence interpretations of behavior, expectations, and classroom management decisions ( Kos et al., 2004; Mohr-Jensen et al., 2019). Limited training and misconceptions may contribute to stigmatizing attributions and less effective management strategies, ultimately affecting students’ academic participation and psychosocial experience at school ( Perold et al., 2009; Mohr-Jensen et al., 2019). Enhancing teachers’ ADHD-related knowledge while fostering supportive attitudes is therefore closely linked to inclusive education and improved outcomes for students with ADHD ( DuPaul & Stoner, 2014; Wolraich et al., 2020). Nevertheless, research using the ASKAT instrument has found that teacher knowledge levels explain only a small proportion of variance in attitudes, indicating that knowledge alone does not necessarily predict supportive or positive attitudes toward students with ADHD. This highlights the importance of measuring and addressing both constructs separately when planning teacher education and professional development ( Mulholland, 2016; Mulholland et al., 2023).

Teachers’ knowledge and attitudes are not consistent across professional groups. Prior research suggests that demographic and professional characteristics—such as gender, educational level, years of experience, and exposure to special or inclusive education training—are associated with variability in ADHD-related knowledge, confidence, and perceptions ( Kos et al., 2004; Greenway & Edwards, 2020). Understanding these differences is relevant for designing targeted professional development and for identifying groups of teachers who may benefit most from ADHD-focused training ( Desimone & Garet, 2015; Darling-Hammond et al., 2017).

To support both research and educational planning, valid and reliable measurement tools are needed to map teachers’ ADHD-related knowledge and attitudes, identify misconceptions and training needs, and evaluate change following professional development initiatives ( DeVellis, 2016; Desimone & Garet, 2015). The ADHD-Specific Knowledge and Attitudes of Teachers (ASKAT) questionnaire was developed to assess knowledge and attitudes within a single instrument and has been used to characterize teacher profiles and training needs ( Mulholland, 2016; Mulholland et al., 2023). However, proper translation, cultural adaptation, and psychometric evaluation are necessary before instruments can be confidently used in new linguistic and educational contexts, especially when the goal is to influence policy and professional development on a large scale ( DeVellis, 2016; Mulholland, 2016). In Greece, there is currently no research that has used the ASKAT tool for preschool and primary school teachers, highlighting the need for psychometric validation of the instrument within the Greek educational context, with particular emphasis on the validation of the SASA subscale ( Mulholland, 2016; Mulholland et al., 2023).

Against this backdrop, the study pursued three objectives: (a) to translate, culturally adapt, and validate ASKAT in Greek, (b) to assess Greek preschool and primary school teachers’ knowledge and attitudes toward ADHD-related behaviors, and (c) to examine demographic and professional correlates of ASKAT scores. These aims provide psychometric evidence for the Greek ASKAT and applied insights relevant to teacher professional development and inclusive practice in Greece, supporting targeted training initiatives and evaluation of training outcomes.

## Methods

### Participants

The sample comprised 1,032 teachers employed in public and private preschool and primary education settings across Greece. Participants were recruited through educational directorates and professional networks. Inclusion criteria included current employment in a teaching position and consent to participate voluntarily. Data collection adhered to ethical research standards, and all procedures were approved by the appropriate institutional review board. The demographic and professional characteristics of the sample are presented in Table 1 (Refer to extended data) ( Boukouvala et al., 2026).

### Research instrument

The ADHD-Specific Knowledge and Attitudes of Teachers (ASKAT) questionnaire was used as the primary instrument. The questionnaire includes four parts:

• **Part A:** Demographic information (e.g., age, gender, teaching level, years of experience, educational background, and training in special/inclusive education).
• **Part B:** ADHD-Specific Knowledge Scale (SASK), comprising 20 true/false items (with an additional “I don’t know” option) assessing teachers’ ADHD-specific knowledge across four domains: symptoms, etiology/causes, treatment, and prevalence.
• **Part C:** ADHD-Specific Attitudes Scale (SASA), which includes Likert-type items categorized into five factors: Negative Consequences, Desire for Professional Learning, Feeling of Knowledgeability, Negative Beliefs, and Positive Consequences.
• **Part D:** Open-ended questions to capture qualitative insights (excluded from the present analysis).

The questionnaire was translated into Greek from the original English version using a forward–backward linguistic adaptation approach. The translation and back-translation were carried out by bilingual individuals, and the final wording was reviewed for clarity and consistency. Minor wording adaptations were made to enhance clarity and ensure cultural and functional appropriateness (online administration; Greek school context), while preserving the conceptual meaning of the items.

### Procedure

The study followed the institution’s Research and Ethics Committee guidelines, obtaining necessary approvals (Approval number: 43508/20.11.2020). Teachers across Greece were informed about the study through email, social media, and online platforms, with the support of regional education directors to promote participation. The questionnaire was distributed through Google Forms and remained open for one year, targeting preschool and primary school teachers, both permanent and substitute, excluding subject-specific teachers such as physical education or music teachers. To avoid duplicate entries, the Google Form was configured to accept only one submission per user account.

The ASKAT questionnaire was translated and culturally adapted into Greek using forward and backward translation methods. Two bilingual experts independently translated the original English version into Greek. This version was then back-translated into English by another bilingual translator. The research team reviewed the resulting materials and resolved any discrepancies, thereby producing a pre-final version. The questionnaire was pilot tested with ten teachers, and minor adjustments were made based on their feedback prior to finalization.

### Statistical analysis

The internal consistency and reliability of the Scale for ADHD-Specific Knowledge (SASK) were assessed using split-half reliability (odd and even items), basic statistical functions, and Cronbach’s alpha, following the procedure outlined by Mulholland (2016). Moreover participants were divided into two groups based on odd or even IDs, and statistical measures (median, mean, etc.) were compared across groups and the overall sample.

Four variables were created to measure the percentage (%) of correct answers in the four following dimensions/subscales: etiology, treatment, symptoms, and prevalence in accordance with the content of SASK questions. T-tests or ANOVA (based on Levene’s test) were used to compare mean percentages between groups, with Bonferroni, LSD, or Tamhane’s T2 applied for multiple comparisons where necessary.

For the Scale for ADHD-Specific Attitudes (SASA), Confirmatory Factor Analysis (CFA) was conducted using R software to evaluate the original five-factor structure (Negative consequences of ADHD, Desire for professional learning, Feeling of Knowledgeability, Negative beliefs about ADHD, and Positive consequences of ADHD). Cronbach’s alpha was used to assess internal consistency for each of the five factors. Group comparisons were conducted using independent-samples t-tests or one-way ANOVA, as appropriate.

Quantitative variables are reported as mean ± standard deviation (SD), and categorical variables as frequencies. A two-tailed p-value <0.05 was considered statistically significant. All analyses, excluding CFA, were conducted using IBM SPSS Statistics version 29. No missing values were present, as all questionnaire items were set as mandatory in the Google Form.

## Results

### ADHD Specific Knowledge (SASK)

Validation was assessed for the Scale for ADHD-Specific Knowledge (SASK), and descriptive statistics were computed across SASK scores and demographic groups.

***Validation of Part B- Scale for ADHD-Specific Knowledge (SASK)***

Following Mulholland’s (2016) procedure, the internal consistency of the Scale for ADHD-Specific Knowledge (SASK) was evaluated. Table 2 (Refer to extended data) ( Boukouvala et al., 2026) presents the split-half results (odd–even items) along with descriptive statistics (mean, mode, standard deviation, skewness, kurtosis, range, minimum, and maximum). Similar values were observed across the three groups. Cronbach’s alpha was 0.656, exceeding the 0.60 threshold suggested by Hair et al. (2010).

Finally, Table 3 (Refer to extended data) ( Boukouvala et al., 2026) presents descriptive statistics (mean, mode, standard deviation, skewness, kurtosis, and minimum–maximum values) for the pooled sample and the two split subsamples (even- and odd-numbered participant IDs). Only slight differences were found among the three groups. These results indicated the consistency and reliability of the Scale for ADHD-Specific Knowledge (SASK).

***Descriptive Statistics of the Part B-Scale for ADHD-Specific Knowledge (SASK)***

Four quantitative variables were computed, quantifying the percentage of correct answers in total and in each of the four dimensions: etiology, treatment, symptoms, and prevalence.

The total mean percentage of correct answers was 78.26 ± 13.09. The mean percentage and standard deviation for each subscale were as follows: Etiology: 65.23 ± 24.1, Treatment: 63.00 ± 23.01, Symptoms: 93.4 ± 11.87, and Prevalence: 73.16 ± 31.17 (Table 4) (Refer to extended data) ( Boukouvala et al., 2026).

***Demographics across ADHD-Specific Knowledge (SASK)***

To assess the mean differences between the (%) score of SASK subscales across demographical characteristics, t-tests or ANOVA (based on Levene’s test) were applied. The study identified several significant findings regarding the impact of demographics on teachers’ mean percentage of correct answers. A statistically significant difference was observed in the mean percentage of correct answers between females and males, with females scoring higher ( *p* = 0.011, two-sided). Special education teachers demonstrated significantly higher mean percentages of correct answers compared to general education teachers (p < 0.001). Similarly, teachers who had received formal special education or Special Education Programs (SEP) achieved significantly higher mean percentages of correct answers compared to those without such training (p < 0.001 for both comparisons).

Additionally, teachers who were not parents outperformed those who were, with the difference approaching statistical significance ( *p* = 0.051, two-sided test). The level of educational attainment also played a crucial role; those with a master’s or doctoral degree achieved significantly higher mean percentages of correct answers compared to teachers holding only a bachelor’s degree (p < 0.001).

Age and years of service were also significant factors. The mean percentage of correct answers varied significantly across different age categories (p = 0.008), with teachers up to 30 years old outperforming those aged 41–50 (p = 0.036). Moreover, the years of service category showed significant differences (p = 0.024), where teachers with 1–5 years of service outperformed those with 21 years of service and above (p = 0.022). These results highlight the influence of various demographic factors on educator performance (Table 5) Refer to extended data ( Boukouvala et al., 2026).

### ADHD Specific Attitudes (SASA)

Following on, validation of ADHD Specific Attitudes was assessed, while descriptive statistics and demographics across SASK subscales were computed.

***Validation of the Part C-Scale for ADHD-Specific Attitudes (SASA)***

*Internal Consistency and Reliability/Confirmatory Analysis – Construct Validity*

Confirmatory factor analysis was conducted to validate the internal structure of factors derived from the application of the same questionnaire in previous studies. The factors included Negative Consequences of ADHD-type Behaviors, Desire for Professional Learning, Feeling of Knowledgeability, Negative Beliefs about ADHD, and Positive Consequences of ADHD-type Behaviors. In Appendix 1, the notation used was provided, along with the R code for obtaining the results and the output. Based on the results in Appendix 1, the Comparative Fit Index (CFI) was 0.961, indicating an excellent fit as it exceeded the recommended threshold of 0.9. The Tucker-Lewis Index (TLI) was 0.953, also indicating a strong fit, surpassing the threshold of 0.9. Furthermore, the Root Mean Square Error of Approximation (RMSEA) was 0.096, slightly above the preferred threshold of 0.08, suggesting a good model fit considering contextual factors such as sample size and model complexity.

As in the study by Mulholland et al. (2023), the CFA included 19 items with the highest factor loadings and strongest conceptual coherence. The remaining 11 items, while not included in the CFA, still hold research value and may be analyzed in future studies with larger samples.

**Figure 1.**
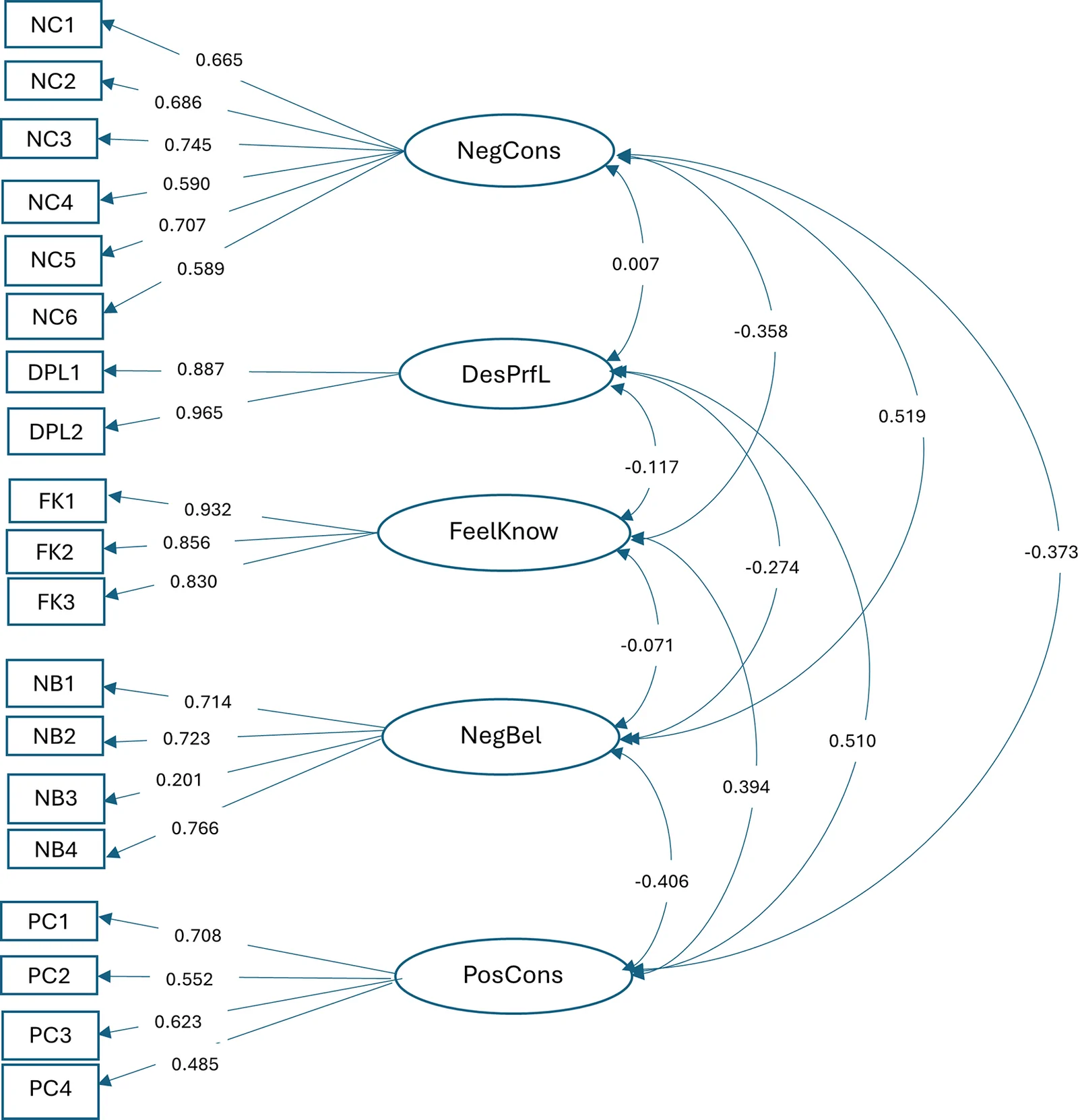
Confirmatory factor analysis of the SASA: the five-factor model structure and item loadings of SASA. NegCons: Negative Consequences; DesPrFL: Desire for Professional learning; FeelKnow Negative consequences of ADHD-type behaviors, Desire for professional learning, FeelKnow: Feeling of knowledgeability; NegBel: Negative beliefs about ADHD; PosCons: Positive consequences of ADHD-type behaviors.

These results indicated that the five-factor structure fit well with the data. Figure 1 illustrates the structure of the five factors, along with the loadings of each question on the corresponding factor and the correlations between the factors. Summarizing, the confirmatory factor analysis of the Scale for ADHD-Specific Attitudes (SASA) aligned with the five-factor structure originally proposed by Mulholland (2016), confirming that this framework remained robust.

Subsequently, Cronbach’s alpha coefficient was used to assess the internal consistency of the items in SASA for each of the five factors mentioned above. Following the approach of Daud et al. (2018), alpha values below 0.6 indicate low reliability, values between 0.6 and 0.8 are considered moderate but acceptable, and values above 0.8 indicate particularly good reliability. The standardized Cronbach’s alpha coefficient for each of the subscales was as follows: Negative Consequences of ADHD-type Behaviors (α = 0.777), Desire for Professional Learning (α = 0.861), Feeling of Knowledgeability (α = 0.876), Negative Beliefs about ADHD (α = 0.612), and Positive Consequences of ADHD-type Behaviors (α = 0.570). It should be noted that the Cronbach’s alpha for Positive Consequences of ADHD-type Behaviors was marginally unsatisfactory.

***Descriptive Statistics of the Part C-Scale for ADHD-Specific Attitudes (SASA)***

The statistical analysis revealed relatively positive Specific Attitudes toward ADHD. The mean scores of beliefs about Negative consequences of ADHD behavior were 13.12 ± 5.08, indicating a moderate attitude. High levels were observed in the desire for professional learning (8.68 ± 1.5) and attitudes toward the positive consequences of ADHD-type behaviors (15.31 ± 2.50). Meanwhile, a moderate level was found for feelings of Knowledgeability (7.50 ± 3.21), and a low level of negative beliefs about ADHD (5.38 ± 3.06) (Table 6) Refer to extended data ( Boukouvala et al., 2026).

***Demographics across ADHD-Specific Attitudes (SASA)***

Το assess the mean differences between the (%) score of SASA subscales across demographical characteristics, t-tests or ANOVA (based on Levene’s test) were applied. Stronger attitudes about Higher scores on *Negative Consequences of ADHD-Type Behaviors* were observed among general education teachers, university graduates, and teachers with special education retraining, compared with postgraduate degree holders and other education categories. Higher scores were also observed among teachers who were parents. In contrast, lower scores on this dimension were observed among younger teachers and those with special education and ADHD-specific training.

In addition, higher Desire for Professional Learning was reported by general education teachers and those without ADHD-specific training. Years of service had a significant impact, with differences observed across experience levels. Teachers with 6–10 years of service had lower scores compared to those with 1–5 years, 11–20 years, and 21+ years. Finally, the impact of administrative positions was noted, with those in sub-managerial roles scoring lower than those in managerial positions.

Regarding the Feeling of Knowledgeability, general education teachers feel decreased confidence in managing ADHD. However, Special education teachers, those with ADHD-specific training and younger teachers feel more knowledgeable. Marital status and years of service also influenced scores (F(2, 166.049) = 22.88, p < 0.001; F(3,1028) = 10.768, p < 0.001 respectively) whereas teachers who were parents reported lower scores (t = −6.727, df = 786.011, p < 0.001) compared to those who were not parents.

Concerning Negative Beliefs about ADHD, it was shown that males, older, those with fewer years of service or lower education levels and those in administrative positions (if they had held a sub-management role) had higher scores indicating more intense negative perceptions. On the contrary, Special education training led to lower scores (t = −2.090, df = 1030, p = 0.037).

About the Positive Consequences of ADHD-Type Behaviors, weak beliefs were shown by the general education teachers, those aged 41–50, those who were parents, and university graduates (compared to postgraduates). Marital status and years of service also affected scores (F(2,1025) = 7.884, p < 0.001; F(3,1028) = 6.180, p < 0.001). Table 7 (Refer to extended data) ( Boukouvala et al., 2026) presents in detail the results of descriptive statistics and correlations with demographic and professional characteristics.

## Discussion

The study aimed to translate, adapt, and validate the ASKAT scale in Greek. It also explored Greek primary teachers’ knowledge and attitudes towards ADHD, examining how demographics and teaching experience affected ASKAT scores.

### Internal consistency analysis of the greek ASKAT questionnaire

Regarding the internal consistency of Part B – Scale for ADHD-Specific Knowledge (SASK), Cronbach’s alpha was calculated at 0.656, exceeding the minimum threshold of 0.60 recommended by Hair et al. (2010). This suggests acceptable reliability for the knowledge scale in the present sample.

In Part C (SASA), the confirmatory factor analysis supported the original five-factor structure proposed by Mulholland (2016), suggesting that the Greek version captures the same underlying attitudinal dimensions (Negative Consequences, Desire for Professional Learning, Feeling of Knowledgeability, Negative Beliefs, and Positive Consequences). Internal consistency was generally acceptable across subscales; however, the “Positive Consequences” factor showed comparatively weaker reliability (α = 0.57). This pattern may reflect the small number of items and limited response variability, but it may also indicate contextual influences—Greek primary teachers may be less familiar with, or less inclined to endorse, positive framings of ADHD-related behaviours. Importantly, the overall structure and reliability profile nevertheless support the SASA as a useful instrument for assessing ADHD-related attitudes, while highlighting “Positive Consequences” as a subscale that may warrant further refinement and validation in larger and more diverse samples. Building on these findings, the psychometric properties of the SASA are discussed below.

This reduced reliability may be attributed to multiple factors: the small number of items in the subscale ( Cortina, 1993), insufficient inter-item correlation ( DeVellis, 2016), and a limited range of participant responses ( Schmitt, 1996). Cultural influences may also play a role, as teachers in the Greek context may be less familiar with or less likely to endorse positive interpretations of ADHD-related behaviors ( Slobodin, 2023). Notably, Mulholland’s (2016) original study reported higher internal consistency for this subscale (α = 0.774), suggesting possible contextual or educational differences between the two populations.

Despite this variation, the internal consistency of the remaining subscales was comparable to that reported by Mulholland, supporting the overall reliability of the SASA in this study. Figure 1 presents a visual representation of the five-factor structure confirmed by the CFA.

### Psychometric properties of SASA

The confirmatory factor analysis supported the original five-factor structure of the SASA, indicating robust construct validity. Most subscales demonstrated satisfactory internal consistency, except for “Positive Consequences,” where lower reliability may reflect cultural factors, limited item number, and reduced inter-item correlation. Nevertheless, the overall scale remains a useful and valid tool for assessing ADHD-related attitudes.

### Assessing knowledge and attitudes of greek teachers

Concerning the ADHD-specific knowledge, the findings highlight significant misconceptions among teachers about ADHD causes, especially related to diet and parenting. While 58.2% answered correctly, many still held incorrect beliefs, suggesting widespread misinformation ( Thapar et al., 2013) and a need for better education on ADHD’s causes and characteristics ( Faraone et al., 2005). In terms of treatment, many teachers mistakenly believe special diets are effective for ADHD, despite scientific evidence against this view ( Sonuga-Barke et al., 2013). The low recognition of this misconception highlights a significant knowledge gap. While over half acknowledge the effectiveness of combined medication and behavioral management, awareness is still limited, suggesting the need for further education ( MTA Cooperative Group, 1999). Most understand that stricter upbringing doesn’t benefit children with ADHD, emphasizing positive support ( Chronis-Tuscano et al., 2011). Additionally, many recognize that children may show ADHD-like behaviors without meeting full diagnostic criteria, reflecting widespread perceptions but also knowledge gaps ( Anastopoulos & Shelton, 2014; Hinshaw & Scheffler, 2014).

In terms of symptoms subscale, the findings of the study highlight widespread recognition among respondents of the primary symptoms exhibited by children with ADHD. For instance, 97.1% acknowledge that children with ADHD often display poor concentration. However, a small percentage of respondents either lack familiarity with the term ADHD or misidentify its associated symptoms. While teachers in this study generally understand the basic symptoms of ADHD, such as inattention and hyperactivity, gaps in their knowledge remain—particularly concerning the disorder’s hereditary nature and the role of diet in managing symptoms. Many teachers are unaware of the genetic factors that contribute to ADHD, which can influence its presentation and severity. Additionally, misconceptions persist regarding the impact of sugar intake and diet on ADHD symptoms, underscoring the need for further education in these areas. Most respondents also noted that improving behavior in children with ADHD is not simply a matter of personal choice, reflecting a broader understanding that these behaviors are shaped by the disorder.

These findings align with previous studies, which similarly indicate that teachers generally have a solid grasp of ADHD’s core symptoms ( Blotnicky-Gallant et al., 2014; Alkahtani, 2013). According to Akatayeva (2022), many of the recent studies on teachers’ knowledge about ADHD ( Cueli et al., 2020; Greenway & Edwards, 2020; Kos et al., 2004; Mulholland et al., 2015; Yarde-Leavett, 2018) have yielded heterogeneous results. Teachers appear to be largely knowledgeable about the basic characteristics of ADHD, such as symptoms or diagnosis. However, there is a significant lack of knowledge in more specific areas related to ADHD, particularly its etiology ( Aguiar et al., 2014; Bekle, 2004; Coronado, 2012; McDougal et al., 2023; Mohr-Jensen et al., 2019; Jones & Chronis-Tuscano, 2008; Kos et al., 2004; Ohan et al., 2008). Moreover, consistent with other research, this study also highlights ongoing gaps in knowledge, particularly around the long-term consequences of ADHD and its impact on behavior, peer relationships, and adherence to classroom rules ( Flanigan and Climie, 2018; Guerra et al., 2017). Finally, the recognition among teachers that ADHD-related behavior is influenced by the disorder rather than personal choice is also in line with earlier studies ( Mulholland, 2016; Sciutto, Terjesen, & Bender Frank, 2000), reinforcing the importance of continued education to help teachers effectively manage ADHD in the classroom.

Regarding the ADHD-specific attitudes, the findings largely align with existing research, though some differences suggest areas needed further exploration. Teachers with special education training demonstrate increased confidence in managing ADHD, consistent with the results of previous studies conducted by DuPaul and Stoner (2014) and Barkley (2015). Similarly, Landolfi (2014) found that such training better equips teachers for the challenges of ADHD, reinforcing the need to integrate special education principles into general education programs.

The results of the present study also support the importance of ongoing support for newly appointed teachers, particularly in inclusive classrooms, as indicated by previous evidence ( Wolraich et al., 2020; Greenhill & Ford, 2002). The desire for continuous professional development among teachers is supported by Mulholland et al. (2023), who found that sustained learning opportunities contribute to improved management of ADHD behaviors. Darling-Hammond et al. (2017) similarly highlighted that teachers engaged in ongoing training tend to be more confident and effective, while they show deeper understanding and empathy toward students with ADHD, promoting inclusivity in the classroom ( Latouche and Gascoigne, 2019).

Regarding the impact of experience and education level on perceptions of ADHD, the findings are consistent with Wehmeier, Schacht, and Barkley (2010) and Kos et al. (2004), highlighting the importance of fostering a positive attitude among teachers and the need for ongoing retraining. Martinussen, Tannock, and Chaban (2011) also highlight the impact of specialized training in reducing misconceptions. Recent research by Ward et al. (2022) emphasizes the crucial role of specialized ADHD training in enhancing teachers’ understanding and perceptions of ADHD.

### Influence of demographic and professional variables

Correlations between demographic characteristics and teachers’ responses are consistent with evidence from other disciplines, suggesting that demographic factors may shape knowledge, beliefs, and attitudes in educational contexts ( Addisu & Abebaw, 2021; Burroughs et al., 2019). With respect to gender, female teachers showed a significantly higher mean percentage of correct answers than male teachers. This pattern accords with studies reporting higher ADHD-related knowledge among female teachers ( Dilaimi, 2013; Jimoh, 2014). However, the literature is mixed. Suleiman (2015) and Safaan et al. (2017) reported no significant gender differences in overall ADHD knowledge (including general information and treatment), although they did identify gender differences in knowledge of symptoms and diagnosis, favouring female teachers. Such inconsistencies across studies may reflect differences in sample characteristics and measurement approaches. As noted by Safaan et al. (2017), one possible interpretation is that gendered roles and experiences—potentially including greater exposure to children’s behavioural and developmental difficulties in everyday contexts—may contribute to greater familiarity with ADHD symptomatology and diagnostic features. Nonetheless, these explanations remain tentative, and the observed gender differences are likely multifactorial.

In line with related work, additional factors that may be associated with gender-linked differences include variability in pedagogical practices, communication styles, and family-related responsibilities ( Islahi & Nasreen, 2013; Luschei, 2012; Sofat, 1977). Overall, these findings highlight the value of considering demographic variation when designing and implementing teacher education and professional development initiatives.

Higher knowledge levels among teachers without children may be partly related to age and more recent exposure to updated ADHD-related training ( Abouammoh et al., 2023). From a cognitive load perspective, parenthood-related demands may also reduce the time and cognitive resources available for continued professional learning ( Braude & Dwarika, 2020; Kennedy & Romig, 2021). At the same time, evidence from the Greek context suggests that parenthood may be associated not only with increased fatigue/burnout, but also with enhanced empathy, communication with parents, and patience ( Koutroumpa et al., 2017).

Moreover, the level of education emerged as a critical factor, with teachers holding master’s or doctoral degrees performing significantly better in ADHD-specific knowledge assessments than those with bachelor’s degrees ( Mohr-Jensen et al., 2019), highlighting the importance of advanced education in enhancing teachers’ knowledge and pedagogical skills, which improve student outcomes ( de Jager et al., 2005; Jacob & Lefgren, 2004).

In addition, teachers with special education training demonstrated a significantly higher mean percentage of correct answers compared to those without such training. This underscores the importance of specialized training in acquiring the knowledge and skills required to address specific challenges in education. Similar findings have been reported in studies by Kos et al. (2004), Mulholland (2016), Sciutto et al. (2000), Stampoltzis and Antonopoulou (2013), and West et al. (2005), which highlighted the influence of varying cultural, educational, and diagnostic factors on ADHD perceptions across different countries.

Research indicates that age and years of service significantly affect teachers’ performance on ADHD knowledge and attitudes assessments. Younger teachers (up to 30 years old) and those with less experience (1–5 years) tend to score higher, possibly due to more recent training and familiarity with modern teaching methods. Conversely, older teachers (41–50 years) and those with over 21 years of experience show a decline in performance, suggesting a need for ongoing professional development. Studies by Amha & Azale (2022), Anderson et al. (2012), and Desimone & Garet (2015) support these findings, emphasizing the importance of continuous education for maintaining effective teaching practices.

This study found no significant link between teachers’ performance and their administrative roles or family status ( Fullan, 1995; Ismail, Arshad, & Abas, 2018). Other research suggests that support and encouragement from school leaders play a crucial role in the implementation of the knowledge and strategies acquired through professional development ( Desimone & Garet, 2015; Loe & Feldman, 2007).

### Implications of the study

This study underscores the need to strengthen the educational framework supporting students with ADHD by equipping teachers with better tools and targeted training. A central contribution is the validation of the Greek ASKAT questionnaire, which reliably assesses teachers’ knowledge and attitudes toward ADHD ( DuPaul & Stoner, 2014; Mulholland et al., 2015). Beyond its research utility, ASKAT could be used in practice by schools or regional education authorities to identify knowledge gaps and design tailored professional development accordingly.

The questionnaire’s data can inform policy and guide the adaptation of instructional strategies to improve learning and behavioral outcomes for students with ADHD ( Avalos, 2011; Wolraich et al., 2020). In addition, it supports the development of evidence-informed teaching practices ( Bryman, 2016; Hattie, 2009).

One key recommendation is to offer structured training programs early in general teachers’ careers, especially for those without prior experience in ADHD-related topics ( Poznanski et al., 2018; Zentall & Javorsky, 2007). Collaboration between general and special education teachers is also essential for promoting inclusive classroom environments ( Velazquez, 2022).

Finally, policymakers should prioritize ADHD awareness in teacher education and consider broader campaigns to support understanding across school communities, emphasizing common challenges like attention and time management, and promoting strategies for effective support ( Chowdhury, 2024; Saad et al., 2022).

### Limitations

Despite its contributions, the present study has certain limitations that should be acknowledged. The focus on preschool and primary education teachers may constrain the extent to which the findings can be generalized to teachers working in other educational contexts, such as secondary or higher education. Future studies incorporating a broader range of educational levels and teaching specialties could provide a more inclusive picture of ADHD-related knowledge and attitudes.

Moreover, the cross-sectional design of the study does not permit inferences regarding causality or developmental changes over time. Longitudinal or mixed-methods approaches would be particularly valuable in capturing shifts in teachers’ knowledge, beliefs, and attitudes toward ADHD across different stages of their professional experience.

While the gender composition of the sample is broadly representative of the teaching workforce in Greece, the predominance of female participants may have limited the visibility of male teachers’ perspectives. Finally, although the ASKAT instrument includes an open-ended section, the qualitative responses were not examined in the present analysis, restricting deeper insight into the reasoning processes and perceptions underlying participants’ answers.

### Strengths

The present study is characterized by several strengths, most notably the inclusion of a large national sample (N = 1,032) of primary school teachers from all educational regions of Greece, which strengthens the robustness of the findings and their potential generalizability. One of the study’s key contributions is the successful translation, cultural adaptation, and validation of the ASKAT questionnaire in Greek. The tool demonstrated solid psychometric properties, particularly in terms of internal consistency and construct validity across most subscales. Additionally, the multifaceted analysis linking demographic and professional variables to both knowledge and attitudes offers valuable insights for designing more targeted training initiatives and informing evidence-based educational policy.

## Conclusions

The ASKAT scale has proven to be a psychometrically valid and reliable tool for evaluating teachers’ knowledge and attitudes toward ADHD in the Greek educational context. This study offers valuable insights into how Greek primary school teachers understand ADHD and respond to ADHD-related behaviors, highlighting both strengths and significant knowledge gaps.

The findings emphasize the urgent need for targeted interventions and structured training programs to address persistent misconceptions—particularly regarding the causes and treatment of ADHD—and to foster more supportive and inclusive attitudes among teachers ( DuPaul & Stoner, 2014; McDougal et al., 2023). The consistent association between higher levels of knowledge and more favorable attitudes further reinforces the importance of integrating ADHD-specific content into both pre-service and in-service teacher education ( Barkley, 2015; Poznanski et al., 2018).

Specialized training and continuous professional development emerged as key factors in equipping teachers with the necessary skills to manage ADHD-related behaviors effectively and empathetically ( Desimone & Garet, 2015; Mulholland et al., 2023). Supporting teachers in understanding the neurodevelopmental nature of ADHD, rather than viewing it through a behavioral or disciplinary lens, is critical to ensuring inclusive and equitable learning environments ( Chowdhury, 2024; Velazquez, 2022).

Finally, by addressing the study’s limitations—such as expanding to secondary education populations and incorporating qualitative analyses—future research can build on these findings to inform evidence-based educational policy and enhance outcomes for students with ADHD in Greece.

## Ethics approval statement

This study was ethically approved by the Research and Ethics Committee (Approval number: 43508/20.11.2020) of University of Ioannina, Ioannina, Greece.

## Informed consent statement

Informed consent was obtained from all participants in written/electronic form via Google Forms prior to questionnaire completion. Participation was voluntary and anonymous, and participants could withdraw at any time without consequences.

## Data Availability

Zenodo: *Dataset for the paper: Psychometric Properties of the Greek Version of the ASKAT: Assessing Teachers’ Knowledge and Attitudes Toward ADHD.* https://doi.org/10.5281/zenodo.18404066 ( Boukouvala et al., 2026). The project contains the following underlying data:
•
**
ASKAT_raw_data_.csv** (De-identified raw questionnaire dataset; original version). •
**
ASKAT_raw_data_English_version.csv** (De-identified raw questionnaire dataset; English-labelled version for readability during peer review). Zenodo: *Dataset for the paper: Psychometric Properties of the Greek Version of the ASKAT: Assessing Teachers’ Knowledge and Attitudes Toward ADHD.* https://doi.org/10.5281/zenodo.18404066 ( Boukouvala et al., 2026). This project contains the following extended data:
• Extended_data.odt (Extended materials supporting the manuscript, including supplementary analyses and documentation). •
Questionnaire_Documentation_with_differences_.odt (Full questionnaire documentation, item wording, labels, and documentation of differences between versions). Data are available under the terms of the Creative Commons Attribution 4.0 International license (CC-BY 4.0).

## References

[ref1] Abouammoh N Younis A Alwatban L : Knowledge about attention deficit hyperactivity disorder among primary school teachers in Riyadh, Saudi Arabia. *J. Nat. Sci. Med.* 2023;6(1):51–57. 10.4103/jnsm.jnsm_22_22

[ref2] Addisu D Abebaw TL : The effects of some selected demographic characteristics on in- service teachers' views of nature of science and process skills. *Brazilian Journal of Education, Technology and Society (Brajets).* 2021. 10.14571/brajets.v14.n3

[ref3] Aguiar AP Kieling RR Costa AC : Increasing teachers' knowledge about ADHD and learning disorders: An investigation on the role of psychoeducational intervention. *J. Atten. Disord.* 2014;18(8):691–698. 10.1177/1087054712453171 22851210

[ref4] Akatayeva A : *Teachers’ knowledge of and attitudes towards ADHD: Nur-Sultan mainstream school teachers’ knowledge of and attitudes towards ADHD.* Nazarbayev University Graduate School of Education;2022. [Master's thesis]. Reference Source

[ref5] Alkahtani KDF : Teachers’ knowledge and misconceptions of attention deficit/hyperactivity disorder. *Psychology.* 2013;04(12):963–969. 10.4236/psych.2013.412139

[ref6] American Psychiatric Association (APA): *Diagnostic and Statistical Manual of mental disorders.* Washington DC: American Psychiatric Association; 5th ed 2013.

[ref7] Amha H Azale T : Attitudes of primary school teachers and its associated factors toward students with attention deficit hyperactivity disorder in Debre Markos and Dejen towns, Northwest Ethiopia. *Front. Pediatr.* 2022;10:805440. 10.3389/fped.2022.805440 35601433 PMC9120593

[ref8] Anastopoulos AD Shelton TL : *Assessing attention-deficit/hyperactivity disorder.* Springer US;2014.

[ref9] Anderson DL Watt SE Noble W : Knowledge of attention deficit hyperactivity disorder (ADHD) and attitudes toward teaching children with ADHD: The role of teaching experience. *Psychol. Sch.* 2012;49(6):511–525. 10.1002/pits.21617

[ref10] Avalos B : Teacher professional development in Teaching and Teacher Education over ten years. *Teach. Teach. Educ.* 2011;27(1):10–20. 10.1016/j.tate.2010.08.007

[ref11] Ayano G Demelash S Gizachew Y : The global prevalence of attention deficit hyperactivity disorder in children and adolescents: An umbrella review of meta-analyses. *J. Affect. Disord.* 2023;339:860–866. 10.1016/j.jad.2023.07.071 37495084

[ref12] Barkley RA : Emotional dysregulation is a core component of ADHD. Barkley RA , editor. *Attention-deficit hyperactivity disorder: A handbook for diagnosis and treatment.* The Guilford Press;2015; pp.81–115.

[ref13] Bekle B : Knowledge and attitudes about attention-deficit hyperactivity disorder (ADHD): A comparison between practicing teachers and undergraduate education students. *J. Atten. Disord.* 2004;7:151–161. 10.1177/108705470400700303 15260172

[ref14] Blotnicky-Gallant P Martin C McGonnell M : Nova Scotia Teachers’ ADHD Knowledge, Beliefs, and Classroom Management Practices. *Can. J. Sch. Psychol.* 2014;30(1):3–21. (Original work published 2015). 10.1177/0829573514542225

[ref15] Boukouvala P Soulis S-G Kotsis K : Dataset for: Psychometric properties of the Greek version of the ASKAT: Assessing teachers’ knowledge and attitudes toward ADHD (Version 2.0).[Dataset]. *Zenodo.* 2026. 10.5281/zenodo.18404066

[ref16] Braude S Dwarika V : Teachers’ experiences of supporting learners with ADHD: Lessons for professional development of teachers. *S Afr J Childhood Educ.* 2020;10(1):a843. 10.4102/sajce.v10i1.843

[ref17] Bryman A : *Social research methods.* Oxford University Press; 5th ed 2016.

[ref18] Burroughs N Gardner J Lee Y : A review of the literature on teacher effectiveness and student outcomes. Burroughs N Gardner J Lee Y , editors. *Teaching for excellence and equity (IEA Research for Education).* Springer;2019; Vol.6: pp.7–17. 10.1007/978-3-030-16151-4_2

[ref19] Chowdhury SA : Developing a Sustainable Education Protocol for Children with Attention Deficit Hyperactivity Disorder (ADHD) in the Context of Developing Country. *J Sci Learn Innov.* 2024;1(1):90–106. 10.1163/29497736-bja00005

[ref20] Chronis-Tuscano A Seymour KE Stein MA : Efficacy of psychosocial treatments for adolescents with ADHD. *J. Clin. Child Adolesc. Psychol.* 2011;40(2):235–262. 10.1016/j.cpr.2006.01.002

[ref21] Coronado CL : *An examination of teachers' knowledge and perceptions of ADHD.* University of Houston–Clear Lake;2012. Doctoral dissertation. Reference Source

[ref22] Cortina JM : What is coefficient alpha? An examination of theory and applications. *J. Appl. Psychol.* 1993;78(1):98–104. 10.1037/0021-9010.78.1.98

[ref24] Cueli M Rodríguez C Cañamero LM : Self-concept and inattention or hyperactivity-impulsivity symptomatology: The role of anxiety. *Brain Sci.* 2020;10(4):250. 10.3390/brainsci10040250 32340167 PMC7226128

[ref25] Cueli M Cañamero LM Rodríguez C : What different education professionals know about ADHD and their attitudes towards it. *Eur. J. Spec. Needs Educ.* 2023;39:33–47. 10.1080/08856257.2023.2185860

[ref26] Darling-Hammond L Hyler ME Gardner M : *Effective teacher professional development.* Learning Policy Institute;2017. Reference Source

[ref27] Daud N Norwani NM Yusof R : Students financial problems in higher education institutions. *International Journal of Academic Research in Business and Social Sciences.* 2018;8(10). 10.6007/IJARBSS/v8-i10/5312

[ref28] Jager B de Jansen M Reezigt G : The development of metacognition in primary school learning environments. *Sch. Eff. Sch. Improv.* 2005;16(2):179–196. 10.1080/09243450500114181

[ref29] Desimone LM Garet MS : Best practices in teachers’ professional development in the United States. *Psychol. Soc. Educ.* 2015;7(3):252–263. 10.25115/psye.v7i3.515

[ref30] DeVellis RF : *Scale development: Theory and applications.* Sage; 4th ed 2016.

[ref31] Dilaimi A : *New Zealand primary school teachers’ knowledge and perceptions of ADHD.* Massey University;2013. Master’s thesis. Reference Source

[ref32] DuPaul GJ Stoner G : *ADHD in the Schools: Assessment and Intervention Strategies.* Guilford Press;2014.

[ref33] Faraone SV Perlis RH Doyle AE : Molecular genetics of attention-deficit/hyperactivity disorder. *Biol. Psychiatry.* 2005;57(11):1313–1323. 10.1016/j.biopsych.2004.11.024 15950004

[ref34] Flanigan L Climie E : Teachers’ knowledge of ADHD: Review and recommendations. *Emerging Perspectives.* 2018;2(1):1–13. Reference Source

[ref35] Fullan MG : The limits and the potential of professional development. Guskey TR Huberman M , editors. *Professional development in education: New paradigms and practices (Chapter 11).* Teachers College Press;1995.

[ref36] Giannopoulou I Korkoliakou P Pasalari E : Greek teachers' knowledge about attention deficit hyperactivity disorder. *Psychiatrike = Psychiatriki.* 2017;28(3):226–233. 10.22365/jpsych.2017.283.226 29072186

[ref38] Greenhill LL Ford RE : Childhood attention-deficit hyperactivity disorder: Pharmacological treatments. Nathan PE Gorman JM , editors. *A guide to treatments that work.* Oxford University Press; 2nd ed. 2002; pp.25–55.

[ref39] Greenway CW Rees Edwards A : Knowledge and attitudes towards attention-deficit hyperactivity disorder (ADHD): a comparison of teachers and teaching assistants. *Australian Journal of Learning Difficulties.* 2020;25(1):31–49. 10.1080/19404158.2019.1709875

[ref40] Guerra F Tiwari A Das A : Examining teachers' understanding of attention deficit hyperactivity disorder. *J. Res. Spec. Educ. Needs.* 2017;17(1):40–48. 10.1111/1471-3802.12382

[ref41] Hair JF Black WC Babin BJ : *Multivariate data analysis: A global perspective.* Pearson;2010.

[ref42] Hattie J : *Visible learning: A synthesis of over 800 meta-analyses relating to achievement.* Routledge;2009.

[ref43] Hinshaw SP Scheffler RM : *The ADHD explosion: Myths, Medication, Money, and Today's Push for Performance.* Oxford University Press;2014.

[ref44] 10.1080/10705519909540118

[ref45] Islahi F Nasreen N : Who make effective teachers, men or women? An Indian perspective. *Univ. J. Educ. Res.* 2013;1(4):285–293. 10.13189/ujer.2013.010402

[ref46] Ismail RAM Arshad R Abas Z : Can Teachers’ Age and Experience influence Teacher Effectiveness in HOTS?. *International Journal of Advanced Studies in Social Science & Innovation (IJASSI).* 2018;2(1). 10.30690/ijassi.21.11

[ref47] Jacob BA Lefgren L : The Impact of Teacher Training on Student Achievement: Quasi-Experimental Evidence from School Reform Efforts in Chicago. *J. Hum. Resour.* 2004;39(1):50–79. 10.3368/jhr.XXXIX.1.50

[ref48] Jimoh M : Knowledge and attitudes towards attention deficit hyperactivity disorder among primary school teachers in Lagos State, Nigeria. *Adv Life Sci Technol.* 2014;23:1–10. 10.7176/ALST

[ref49] Jones HA Chronis-Tuscano A : Efficacy of teacher in-service training for attention-deficit/hyperactivity disorder. *Psychol. Sch.* 2008;45(10):918–929. 10.1002/pits.20342

[ref50] Kennedy MJ Romig JE : Cognitive load theory: An applied reintroduction for special and general educators. *Teach. Except. Child.* 2021;56:440–451. 10.1177/00400599211048214

[ref51] Kooij JJS Bijlenga D Salerno L : Updated European consensus statement on diagnosis and treatment of adult ADHD. *Eur. Psychiatry.* 2019;56:14–34. 10.1016/j.eurpsy.2018.11.001 30453134

[ref52] Kos JM Richdale AL Jackson MS : Knowledge About Attention-Deficit/Hyperactivity Disorder: A Comparison of In-Service and Preservice Teachers. *Psychol. Sch.* 2004;41(5):517–526. 10.1002/pits.10178

[ref53] Koutroumpa K Kalantzaki E Christopoulos I : When teachers become parents: Parenthood influence on Greek teachers’ professional lives. *Eur. j. soc. sci. educ. res.* 2017;4(1):20–30. 10.26417/ejser.v7i1.p20-30

[ref55] Landolfi A-M : *Inclusive classroom communities: Supporting students with characteristics of attention deficit hyperactivity disorder.* Ontario Institute for Studies in Education of the University of Toronto. TSpace;2014. (Master’s research paper). Reference Source

[ref56] Latouche AP Gascoigne M : In-Service Training for Increasing Teachers’ ADHD Knowledge and Self-Efficacy. *J. Atten. Disord.* 2019;23(3):270–281. 10.1177/1087054717707045 28494656

[ref57] Loe IM Feldman HM : Academic and educational outcomes of children with ADHD. *J. Pediatr. Psychol.* 2007;32(6):643–654. 10.1093/jpepsy/jsl054 17569716

[ref58] Luschei TF : The effectiveness and distribution of male primary teachers: Evidence from two Mexican states. *Int. J. Educ. Dev.* 2012;32(1):145–154. 10.1016/j.ijedudev.2010.12.001

[ref59] Martinussen R Tannock R Chaban P : Teachers’ reported use of instructional and behavior management practices for students with behavior problems: Relationship to role and level of training in ADHD. *Child Youth Care Forum.* 2011;40(3):193–210. 10.1007/s10566-010-9130-6

[ref60] McDougal E Tai C Stewart TM : Understanding and supporting attention deficit hyperactivity disorder (ADHD) in the primary school classroom: Perspectives of children with ADHD and their teachers. *J. Autism Dev. Disord.* 2023;53:3406–3421. 10.1007/s10803-022-05639-3 35776263 PMC10465390

[ref61] Mohr-Jensen C Steen-Jensen T Bang-Schnack M : What Do Primary and Secondary School Teachers Know About ADHD in Children? Findings From a Systematic Review and a Representative, Nationwide Sample of Danish Teachers. *J. Atten. Disord.* 2019;23(3):206–219. 10.1177/1087054715599206 26297913

[ref62] MTA Cooperative Group: A 14-month randomized clinical trial of treatment strategies for attention-deficit/hyperactivity disorder. *Arch. Gen. Psychiatry.* 1999;56(12):1073–1086. 10.1001/archpsyc.56.12.1073 10591283

[ref63] Mulholland SM Cumming TM Jung JY : Teacher attitudes towards students who exhibit ADHD-type behaviours. *Australasian Journal of Special Education.* 2015;39(1):15–36. 10.1017/jse.2014.18

[ref64] Mulholland S : ADHD-specific knowledge and attitudes of teachers (ASKAT): Development and validation of a new research instrument. *Int. J. Educ. Res.* 2016;77:109–116. 10.1016/j.ijer.2016.03.010

[ref65] Mulholland S Cumming TM Lee J : Accurately Assessing Teacher ADHD-Specific Attitudes Using the Scale for ADHD-Specific Attitudes. *J. Atten. Disord.* 2023;27(5):554–568. 10.1177/10870547231153938 36843350 PMC9978867

[ref66] Ohan JL Cormier N Hepp SL : Does knowledge about attention-deficit/hyperactivity disorder impact teachers’ reported behaviors and perceptions?. *Sch. Psychol. Q.* 2008;23(3):436–449. 10.1037/1045-3830.23.3.436

[ref67] Perold M Louw C Kleynhans S : Primary school teachers' knowledge and misperceptions of attention deficit hyperactivity disorder (ADHD). *South African J. Educ.* 2009;30(3). 10.4314/saje.v30i3.60041

[ref68] Poznanski B Hart KC Cramer E : Are teachers ready? Preservice teacher knowledge of classroom management and ADHD. *Sch. Ment. Heal.* 2018;10(3):301–313. 10.1007/s12310-018-9259-2

[ref69] Rajaprakash M Leppert ML : Attention-Deficit/Hyperactivity Disorder. *Pediatr. Rev.* 2022,2022 Mar 1;43(3):135–147. 10.1542/pir.2020-0006 35229109

[ref70] Saad S Aljanahi F Coumaravelou S : Knowledge about attention-deficit/hyperactivity disorder among primary schoolteachers in Sharjah, UAE. *J. Educ. Health Promot.* 2022;11(1):99. 10.4103/jehp.jehp_957_21 35573627 PMC9093637

[ref71] Safaan NA El-Nagar SA Saleh AG : Teachers' knowledge about Attention Deficit Hyperactivity Disorder among primary school children. *American J. Nurs. Res.* 2017;5(2):42–52. 10.12691/ajnr-5-2-2

[ref72] Salari N Ghasemi H Abdoli N : The global prevalence of ADHD in children and adolescents: A systematic review and meta-analysis. *Ital. J. Pediatr.* 2023;49:48. 10.1186/s13052-023-01456-1 37081447 PMC10120242

[ref73] Sciutto MJ Terjesen MD Bender Frank AS : Teachers' knowledge and misperceptions of Attention-Deficit/Hyperactivity Disorder. *Psychol. Sch.* 2000;37(2):115–122.

[ref74] Schmitt N : Uses and abuses of coefficient alpha. *Psychol. Assess.* 1996;8(4):350–353. 10.1037/1040-3590.8.4.350

[ref76] Slobodin O : ADHD in culturally and linguistically diverse children. *Clinical handbook of ADHD assessment and treatment across the lifespan.* Springer International Publishing;2023; pp.1–15. 10.1007/978-3-031-41709-2_1

[ref77] Sofat SL : Construction and standardization of self-evaluation scale of teaching effectiveness of secondary school teachers. *Fourth Survey of Educational Research.* 1977;II:1041.

[ref78] Sonuga-Barke EJ Brandeis D Cortese S : Nonpharmacological interventions for ADHD: Systematic review and meta-analyses of randomized controlled trials of dietary and psychological treatments. *Am. J. Psychiatry.* 2013;170(3):275–289. 10.1176/appi.ajp.2012.12070991 23360949

[ref79] Stampoltzis A Antonopoulou K : Knowledge and misconceptions about Attention Deficit Hyperactivity Disorder (ADHD): A comparison of Greek general and special education teachers. *Int. J. Sch. Educ. Psychol.* 2013;1(2):122–130. 10.1080/21683603.2013.803000

[ref80] Suleiman ME : Primary school teachers’ knowledge of ADHD in Beni-Suef governorate in Egypt. *The Islamic University of Educational and Psychological Studies Journal.* 2015;23(1):98–121.

[ref81] Thapar A Cooper M Eyre O : What have we learnt about the causes of ADHD?. *J. Child Psychol. Psychiatry.* 2013;54(1):3–16. 10.1111/j.1469-7610.2012.02611.x 22963644 PMC3572580

[ref82] Ward RJ Bristow SJ Kovshoff H : The effects of ADHD teacher training programs on teachers and pupils: A systematic review and meta-analysis. *J. Atten. Disord.* 2022;26(2):225–244. 10.1177/1087054720972801 33331193 PMC8679179

[ref83] Velazquez K : *Collaborative networks of general and special education teachers at an inclusive school site.* University of California, San Diego;2022. (Doctoral dissertation). **ProQuest Dissertations & Theses Global**.

[ref84] Wehmeier PM Schacht A Barkley RA : Social and Emotional Impairment in Children and Adolescents with ADHD and the Impact on Quality of Life. *J. Adolesc. Health.* 2010;46(3):209–217. 10.1016/j.jadohealth.2009.09.009 20159496

[ref85] West J Taylor M Houghton S : A Comparison of Teachers’ and Parents’ Knowledge and Beliefs About Attention-Deficit/Hyperactivity Disorder (ADHD). *Sch. Psychol. Int.* 2005;26(2):192–208. (Original work published 2005). 10.1177/0143034305052913

[ref86] Wolraich ML Hagan JF Jr Allan C : Erratum: Clinical practice guideline for the diagnosis, evaluation, and treatment of attention-deficit/hyperactivity disorder in children and adolescents. *Pediatrics.* 2020;145(3):e20193997. 10.1542/peds.2019-3997 32111626

[ref88] Yarde-Leavett C : *Teachers’ knowledge of and attitudes towards ADHD in the Western Cape.* University of Cape Town, Department of Psychology;2018. (master’s thesis).

[ref89] Zentall SS Javorsky J : Professional Development for Teachers of Students with ADHD and Characteristics of ADHD. *Behav. Disord.* 2007;32(2):78–93. 10.1177/019874290703200202

