# Supplementary material for: Psychometric Properties of the Greek Version of the ASKAT: Assessing Teachers’ Knowledge and Attitudes Toward ADHD
Source: F1000Res. 2026 Jul 11;15:509. Originally published 2026 Apr 13. [Version 2] doi: 10.12688/f1000research.176376.2 (PMC13554785; doi:10.12688/f1000research.176376.2)
Supplement: Peer review history

# Reviewer report: Reviewer response for version 2

**DOI:** 10.5256/f1000research.203401.r500519

**Published:** 2026-07-16

**Authors:** Kalliopi Trouli

**Affiliations:** 1 University of Crete, Crete, Greece

I have carefully reviewed the revised version of the manuscript entitled *"Psychometric Properties of the Greek Version of the ASKAT: Assessing Teachers' Knowledge and Attitudes Toward ADHD."*

 I am pleased to confirm that the authors have thoroughly addressed the comments and recommendations raised during the previous review phase. The revisions substantially strengthen the methodological transparency, scientific rigor, and overall clarity of the manuscript. The study makes a valuable contribution to the field by providing a validated Greek version of the ASKAT questionnaire, derived from a large nationwide sample, which constitutes a significant resource for future research on teacher readiness regarding ADHD in Greek-speaking educational contexts.

 I am confident that the manuscript now meets the scientific and methodological standards required for indexing and recommend acceptance in its present form.

Is the work clearly and accurately presented and does it cite the current literature?

Yes

If applicable, is the statistical analysis and its interpretation appropriate?

Yes

Are all the source data underlying the results available to ensure full reproducibility?

Yes

Is the study design appropriate and is the work technically sound?

Yes

Are the conclusions drawn adequately supported by the results?

Yes

Are sufficient details of methods and analysis provided to allow replication by others?

Partly

Reviewer Expertise:

My research focuses on psychomotor education, physical education in early childhood settings, and the development of graphomotor skills in kindergarten children. A significant part of my work involves the development and psychometric validation of assessment instruments for preschool education.

I confirm that I have read this submission and believe that I have an appropriate level of expertise to confirm that it is of an acceptable scientific standard.

# Reviewer report: Reviewer response for version 2

**DOI:** 10.5256/f1000research.203401.r500520

**Published:** 2026-07-15

**Authors:** Constantinos Togas

**Affiliations:** 1 Hellenic Open University, Athens, Greece

Dear Editor,

 I have carefully reviewed the revised version of this manuscript. The authors have adequately addressed all the issues raised in my initial review. The manuscript has been substantially improved, and I believe it is now suitable for indexing in its current form.

 Best regards

 C.T.

Is the work clearly and accurately presented and does it cite the current literature?

Yes

If applicable, is the statistical analysis and its interpretation appropriate?

Yes

Are all the source data underlying the results available to ensure full reproducibility?

Yes

Is the study design appropriate and is the work technically sound?

Yes

Are the conclusions drawn adequately supported by the results?

Yes

Are sufficient details of methods and analysis provided to allow replication by others?

Partly

Reviewer Expertise:

NA

I confirm that I have read this submission and believe that I have an appropriate level of expertise to confirm that it is of an acceptable scientific standard.

# Reviewer report: Reviewer response for version 1

**DOI:** 10.5256/f1000research.194425.r481342

**Published:** 2026-07-08

**Authors:** Kalliopi Trouli

**Affiliations:** 1 University of Crete, Crete, Greece

General Evaluation

 I have carefully reviewed the manuscript, which presents the translation, cross-cultural adaptation, and psychometric validation of the ADHD-Specific Knowledge and Attitudes of Teachers (ASKAT) questionnaire into Greek. I have also thoroughly examined the comments and recommendations provided by the first reviewer. I fully concur with the first reviewer's observations and consider it essential that all suggested modifications be incorporated into the final manuscript.

 The study demonstrates notable strengths, including a large nationwide sample of preschool and primary education teachers (N = 1.032), and addresses a significant gap in the Greek educational literature regarding the assessment of teacher readiness concerning ADHD.

 While the study makes a valuable contribution to the field, I have identified additional points that require revision to strengthen the methodological transparency and scientific rigor of the manuscript.

 Specific Comments and Recommendations

 Research Instrument Section (Part C)

 The description of the SASA scale structure requires clarification. Specifically, the authors should explicitly state which items were used in the psychometric analysis and how subscale scores were calculated. This clarification is necessary to prevent potential reader confusion regarding the correspondence between the total number of scale items and those included in the factor-analytic procedures.

 Procedure Section

 Comment 1: The procedure section requires a more detailed description of the translation and cross-cultural adaptation methodology. The authors should provide a comprehensive description of the forward-backward translation method employed, as well as the pilot testing procedure conducted with teachers and the refinements that emerged from this process. This information is essential, as thorough documentation of the cross-cultural adaptation procedure constitutes a fundamental requirement in translation and validation studies of psychometric instruments.

 Comment 2: The statistical strategy for validating the SASA scale requires further elaboration. I recommend that the authors include a clear description of the Confirmatory Factor Analysis (CFA) conducted to test the five-factor structure in the Greek population. This information is necessary to clearly establish the approach used for assessing construct validity and reliability estimation.

 Discussion Section

 The interpretation of psychometric findings, particularly regarding the internal consistency analysis of the Greek ASKAT questionnaire, requires expansion. The authors should comment on the internal consistency of both the SASK and SASA scales and provide a rationale for the lower reliability observed in the "Positive Consequences" subscale, drawing on relevant literature and possible cultural or educational characteristics of the Greek sample. This addition would enhance the scientific rigor and transparency of the study.

 Limitations Section

 The limitations section should include reference to the relatively low internal consistency of the "Positive Consequences" subscale. Acknowledgment of this psychometric limitation would enhance the transparency and scientific integrity of the manuscript.

 Recommendation

 The manuscript is approved with reservations.

Is the work clearly and accurately presented and does it cite the current literature?

Yes

If applicable, is the statistical analysis and its interpretation appropriate?

Yes

Are all the source data underlying the results available to ensure full reproducibility?

Yes

Is the study design appropriate and is the work technically sound?

Yes

Are the conclusions drawn adequately supported by the results?

Yes

Are sufficient details of methods and analysis provided to allow replication by others?

Partly

Reviewer Expertise:

My research focuses on psychomotor education, physical education in early childhood settings, and the development of graphomotor skills in kindergarten children. A significant part of my work involves the development and psychometric validation of assessment instruments for preschool education.

I confirm that I have read this submission and believe that I have an appropriate level of expertise to confirm that it is of an acceptable scientific standard, however I have significant reservations, as outlined above.

# Response

**Published:** 2026-07-12

**Authors:** ΠΑΡΑΣΚΕΥΗ ΜΠΟΥΚΟΥΒΑΛΑ

Dear Reviewer,

 We would like to thank you for your careful reading of our manuscript and for your constructive comments. We would like to clarify that your review concerns Version 1 of the manuscript. Following the first round of peer review, the manuscript was revised and Version 2 was subsequently published. Having carefully considered the points raised in your report in relation to the revised manuscript, we found that the modifications incorporated into Version 2 address all of your recommendations. Below, we provide a point-by-point response.

**Comment 1: Research Instrument Section (Part C). The description of the SASA scale structure requires clarification, particularly regarding the items included in the psychometric analysis and the calculation of the subscale scores.**

 In Version 2, the “Research Instrument” subsection was expanded to clarify that all 30 SASA items were administered, while the CFA and the calculation of the five subscale scores were based on the 19 items included in the five-factor structure identified by Mulholland (2016). The number of items assigned to each factor and the scoring procedure are also explicitly described.

**Comment 2: Procedure Section – Translation and Cross-Cultural Adaptation. A more detailed description of the forward–backward translation procedure, pilot testing, and resulting refinements is required.**

 The revised “Procedure” subsection provides a detailed description of the independent forward translations, back-translation, comparison and resolution of discrepancies, and development of the pre-final Greek version. It also describes the pilot testing conducted with ten preschool and primary school teachers and the minor linguistic and formatting adjustments that resulted from their feedback.

**Comment 3: Procedure Section – Statistical Strategy for the SASA. The statistical strategy used to validate the five-factor structure of the SASA through Confirmatory Factor Analysis should be described more clearly.**

 The revised “Statistical analysis” subsection clearly states that CFA was conducted in R to examine the five-factor structure originally identified by Mulholland (2016) in the Greek sample. It specifies the 19 items included in the model, the five correlated latent factors, and the calculation of Cronbach’s alpha for each corresponding subscale. The relevant fit indices, R code, and output are also reported.

**Comment 4: Discussion Section. The interpretation of the internal consistency findings for the SASK and SASA should be expanded, with particular attention to the lower reliability of the “Positive Consequences” subscale.**

 The revised Discussion provides a more detailed interpretation of the internal consistency findings for both the SASK and the five SASA subscales. It also discusses possible explanations for the lower reliability of the “Positive Consequences” subscale, including the limited number of items, inter-item correlations, response variability, and possible cultural and educational influences within the Greek context. The findings are also compared with those reported in the original validation study conducted by Mulholland (2016).

**Comment 5: Limitations Section. The relatively low internal consistency of the “Positive Consequences” subscale should be acknowledged as a limitation.**

 The revised “Limitations” section explicitly acknowledges the comparatively weak internal consistency of the “Positive Consequences” subscale and recommends its further examination and refinement in larger and more diverse educational and cultural samples.

 We believe that the revisions incorporated into Version 2 address all the comments and recommendations included in the present review. We sincerely thank the reviewer for the positive evaluation of the study and for the valuable recommendations.

 Sincerely,

 The authors

# Reviewer report: Reviewer response for version 1

**DOI:** 10.5256/f1000research.194425.r478997

**Published:** 2026-06-04

**Authors:** Constantinos Togas

**Affiliations:** 1 Hellenic Open University, Athens, Greece

I carefully reviewed the manuscript and found it to be of considerable interest. This is a well-designed study with important strengths and implications for the Greek population. Its strengths include originality in the Greek population and the relatively large sample size. Please find my detailed comments below.

**Abstract – Methods:**

 Please specify the study period and the overall duration of the study. In addition, please indicate that the questionnaire was administered online through Google Forms.

**Introduction – Description of the Questionnaire:**

**The description of the questionnaire in the Introduction could be expanded to provide a more comprehensive overview of the instrument. Specifically, the authors may consider including information on its factor structure, the psychometric properties reported in the original validation study, and whether the instrument has been translated and validated in other languages or cultural settings. If so, a brief summary of the psychometric findings from these studies would be valuable. In addition, it would be helpful to mention whether the questionnaire has been used in previous research and to provide examples of relevant applications.**

**Research Instrument:**

 The separate description of the two questionnaire subscales may be confusing for readers. I suggest presenting the instrument in a dedicated subsection that includes the following information: the total number of items, the subscales, the number of items per subscale, example items, the scoring procedure, and the interpretation of the scores (including what a higher score indicates).

 Research instrument

*“…..The questionnaire was translated into Greek from the original English version using a forward–backward linguistic adaptation approach. The translation and back-translation were carried out by bilingual individuals, and the final wording was reviewed for clarity and consistency. Minor wording adaptations were made to enhance clarity and ensure cultural and functional appropriateness (online administration; Greek school context), while preserving the conceptual meaning of the items…*..”    =   The translation process is also presented later in the subsection entitled 'Procedure'.

**Methods Participants**

 The statement "Data collection adhered to ethical research standards, and all procedures were approved by the appropriate institutional review board" could be moved to the Procedure subsection, as ethical considerations and approval procedures are described there. This would improve the organization of the manuscript and avoid redundancy.

 page. 6, Cronbach’s alpha coefficient = The authors can add  Cronbach’s alpha coefficient if item deleted

 The authors may consider adding a table presenting descriptive statistics for each questionnaire item. Such a table could include the mean, standard deviation, minimum and maximum values, range, and median for each item. Providing item-level descriptive statistics would offer readers a more detailed understanding of response patterns and the distribution of scores across the questionnaire.

 If the journal follows APA Style 7, in-text citations should be arranged alphabetically when multiple references are cited within the same parentheses.

 There is also a large number of references, which should be reduced and summarized to a maximum of 40.

Is the work clearly and accurately presented and does it cite the current literature?

Yes

If applicable, is the statistical analysis and its interpretation appropriate?

Yes

Are all the source data underlying the results available to ensure full reproducibility?

Yes

Is the study design appropriate and is the work technically sound?

Yes

Are the conclusions drawn adequately supported by the results?

Yes

Are sufficient details of methods and analysis provided to allow replication by others?

Partly

Reviewer Expertise:

NA

I confirm that I have read this submission and believe that I have an appropriate level of expertise to confirm that it is of an acceptable scientific standard, however I have significant reservations, as outlined above.

# Response

**Published:** 2026-07-01

**Authors:** ΠΑΡΑΣΚΕΥΗ ΜΠΟΥΚΟΥΒΑΛΑ

Dear Reviewer,

 We would like to thank you for your careful reading of our manuscript and for your constructive comments. We have revised the manuscript in response to your suggestions, with the aim of improving its clarity, organization, methodological transparency, and reproducibility. Below, we provide a point-by-point response:

**Comment 1: Abstract – Methods. Please specify the study period and the overall duration of the study. In addition, please indicate that the questionnaire was administered online through Google Forms.**

 We revised the Methods section of the Abstract to specify that data collection was conducted from May 2022 to May 2023, over an approximate 12-month period. We also clarified that the ASKAT questionnaire was administered online through Google Forms.

**Comment 2: Introduction – Description of the Questionnaire. The description of the questionnaire in the Introduction could be expanded to provide a more comprehensive overview of the instrument.**

 The Introduction has been expanded to provide a clearer overview of the ASKAT questionnaire. We added information on its two main quantitative components, namely the Scale for ADHD-Specific Knowledge (SASK) and the Scale of ADHD-Specific Attitudes (SASA). We also included further information on the SASA factor structure, the psychometric evidence reported in the original validation study, and subsequent research using the instrument. This addition aims to help readers better understand the structure, purpose, and previous use of the questionnaire.

**Comment 3: Research Instrument. The separate description of the two questionnaire subscales may be confusing for readers.**

 We revised the “Research Instrument” subsection to present the ASKAT more clearly and coherently. The revised subsection now explains that the SASK and the SASA are two distinct quantitative components of the ASKAT, assessing different constructs: ADHD-specific knowledge and ADHD-specific attitudes, respectively. We clarified that these components can be scored, interpreted, and used separately depending on the aims of each study, while both were administered in the present study as part of the Greek adaptation and psychometric evaluation of the ASKAT. The revised subsection also includes the total number of scored quantitative items, the subscales, the number of items per scale/subscale, the scoring procedure, and the interpretation of higher scores. We also clarified that the open-ended questions were not included in the present quantitative analyses.

**Comment 4: Research Instrument. The translation process is also presented later in the subsection entitled “Procedure”.**

 We removed the repeated description of the translation process from the “Research Instrument” subsection in order to avoid redundancy. The translation and cultural adaptation procedure is now presented only in the “Procedure” subsection, where it is described in a more organized way.

**Comment 5: Methods – Participants. The statement “Data collection adhered to ethical research standards, and all procedures were approved by the appropriate institutional review board” could be moved to the Procedure subsection.**

 We moved the statement regarding ethical standards and institutional approval from the “Participants” subsection to the “Procedure” subsection. This section now presents the ethical approval, informed consent, anonymity, and confidentiality procedures more coherently.

**Comment 6: Page 6, Cronbach’s alpha coefficient. The authors can add Cronbach’s alpha coefficient if item deleted.**

 We calculated Cronbach’s alpha if item deleted and added the results to the Extended data. For the SASK, Cronbach’s alpha if item deleted was calculated for each of the 20 knowledge items and is now presented in Table 8. For the SASA, Cronbach’s alpha if item deleted was calculated separately for the items included in each corresponding SASA factor/subscale and is now presented in Table 9. We also clarified that Cronbach’s alpha if item deleted was not applicable for the Desire for Professional Learning factor, as this factor includes only two items. These additions provide further item-level reliability evidence for both components of the ASKAT.

**Comment 7: The authors may consider adding a table presenting descriptive statistics for each questionnaire item.**

 We added item-level descriptive statistics for the scored ASKAT items to the Extended data. Specifically, item-level descriptive statistics for the SASK items are now presented in Table 10, including the mean, standard deviation, median, minimum and maximum values, range, and percentage of correct responses. Since SASK items were coded as 1 = correct and 0 = incorrect or “I don’t know”, the mean reflects the proportion of correct responses for each item. Item-level descriptive statistics for the scored SASA items are now presented in Table 11, including the mean, standard deviation, median, minimum and maximum values, and range. These additions improve the transparency and reproducibility of the descriptive results.

**Comment 8: If the journal follows APA Style 7, in-text citations should be arranged alphabetically when multiple references are cited within the same parentheses.**

 We reviewed the manuscript and corrected the order of multiple in-text citations within the same parentheses according to APA Style 7, arranging them alphabetically where required.

**Comment 9: There are also many references, which should be reduced and summarized to a maximum of 40.**

 We reviewed the reference list and removed redundant or less central references where possible. At the same time, we retained key references that were necessary for supporting the theoretical background, the ASKAT instrument, the psychometric validation procedures, and the interpretation of the findings. Since F1000Research does not appear to specify a strict maximum of 40 references for Research Articles, we aimed to reduce the reference list in a balanced way without removing sources that are essential for the scientific documentation of the study.

 We believe that these revisions have improved the clarity, structure, and methodological transparency of the manuscript.

 Sincerely,

 The authors
